# Investigating Multiple Streams of Consciousness: Using Descriptive Experience Sampling to Explore Internally and Externally Directed Streams of Thought

**DOI:** 10.3389/fnhum.2018.00494

**Published:** 2018-12-06

**Authors:** Charles Fernyhough, Ben Alderson-Day, Russell T. Hurlburt, Simone Kühn

**Affiliations:** ^1^Department of Psychology, Durham University, Durham, United Kingdom; ^2^Department of Psychology, University of Nevada, Las Vegas, Las Vegas, NV, United States; ^3^Center for Lifespan Psychology, Max Planck Institute for Human Development, Berlin, Germany; ^4^Department of Psychiatry and Psychotherapy, University Medical Center Hamburg-Eppendorf, Hamburg, Germany

**Keywords:** resting state, fMRI, default mode network, frontal-parietal network, dorsal attention network, stimulus-independent thought, mind-wandering

## Abstract

Research into resting-state cognition has often struggled with the challenge of assessing inner experience in the resting state. We employed Descriptive Experience Sampling (DES), a method aimed at generating detailed and high-fidelity descriptions of experience, to investigate how experience in the resting state can vary between internal, external, and multiple simultaneous streams. Using a large body of experiential and brain activation data acquired from five DES participants, independent raters classified sampled moments of experience according to whether they were internally directed, externally directed, or contained elements of both at the same time. In line with existing models, comparison of internal with external experience samples identified a network of regions associated with the default mode network. Regions of interest resulting from the whole-brain contrasts successfully predicted independent raters’ forced-choice categorizations of samples for which experience had a simultaneous internal and external focus. The present study is distinctive in tying neural activations in the resting state to detailed descriptions of specific phenomenology, and in demonstrating how the DES method enables a particularly nuanced analysis of moments of experience, especially their ability simultaneously to incorporate both an internal and an external focus. The study represents an integration of rich phenomenology and characterizations of brain activity, tracing interpretive paths from phenomenology to neural activation and *vice versa*.

## Introduction

There has been a significant growth of interest in studying the brain when participants are not engaged in any particular task (so-called resting-state measurements). An increased understanding of resting-state brain networks has proved valuable for theorizing about a range of psychological phenomena, including autobiographical memory (Spreng et al., 2009), social cognition (Spunt et al., 2015), and hallucinations (Alderson-Day et al., 2016). Resting-state studies report brain activity in a consistent network of regions, including lower precuneus, superior and inferior anterior medial frontal regions, and posterior lateral parietal cortices (Gusnard and Raichle, 2001). The consistency with which activity in these brain regions decreases during tasks and increases during rest has led to the notion of a so-called “default mode” network (DMN) of the brain (Buckner et al., 2008).

This growth of interest in the brain’s resting-state networks has been paralleled by an increase of interest in cognitive processes that are not stimulated by any particular external stimuli, commonly referred to as mind-wandering (Callard et al., 2013). To date, efforts to link such “stimulus-independent” cognitions to their underlying neural states have been hampered by the difficulty of capturing descriptions of ongoing cognitive states in sufficient fidelity, richness, and detail.

One method for assessing subjective experience in the scanner involves asking participants to fill in a questionnaire after completing a resting-state scan, with the intention of characterizing the general qualities of their experience while in the scanner (Delamillieure et al., 2010). However, the complex, multimodal, and dynamic patterning of the stream of consciousness is likely to vary considerably in its qualities from moment to moment within one resting-state scan, as well as from scan to scan and from individual to individual. Furthermore, resting-state questionnaires (like all questionnaires) are limited by the particular questions they ask and the particular way in which they ask them. As a result, such retrospective attempts to describe general qualities of a relatively long period of consciousness are not likely to provide accurate or particularly detailed descriptions of experience in the resting state (Hurlburt et al., 2015; Van Calster et al., 2017).

Some have sought to overcome the retrospectiveness problem by presenting random probes and soliciting reports immediately thereafter. For example, Christoff et al. (2009) had participants in the scanner perform a boring task and, when signaled, report whether their attention was focused on the task or otherwise. However, while ameliorating the retrospectiveness problem, this quantitative probing method has shortcomings of its own, principally that the response options available in such a paradigm are highly constrained: for example, the Christoff et al. (2009) participants responded only to two Likert-type scales, one asking the degree to which attention was focused on the task and the other whether they had been aware of where their attention was focused. Raij and Riekki (2017) recently employed a more sophisticated probe methodology to explore neural activations during spontaneous thinking, with responses based on an answer tree derived from previous research. However, the response procedure was complex (involving two separate button-presses) and probes occurred at fairly frequent intervals (between 12 and 40.5 s apart), meaning that the resting state was frequently disrupted by a probe. In all such studies, it is possible that a participant’s responses on such self-report scales may depend more on presuppositions about experience than on experience itself (Hurlburt and Heavey, 2015).

Methodological limitations like these have meant that the phenomenology of resting-state cognition has not been described in detail, and that key questions about resting-state cognition remain largely unanswered. For example, some have proposed that the alternation between task-centered cognition and mind-wandering involves an organized and periodic switching between neural networks involved in processing information from the environment and those that manipulate internally generated information (Spreng et al., 2010, 2013; Smallwood et al., 2012). If this is the case, it should be reflected in the phenomenology of the resting-state experience, and it should in principle be possible to measure concurrent changes to areas of the DMN and other networks during moments of internally and externally guided attention.

One focus of such studies has been to model how the human cognitive system can move between psychological states that are focused on the external environment and those that involve internal generation of material. Of particular interest is the positing of a decoupling mechanism that frees the agent from acting solely on immediate, environmentally triggered stimuli. Several authors (Spreng et al., 2010, 2013; Smallwood et al., 2012) have proposed a model of stimulus-independent thought that depends on cooperation between the DMN (which provides autobiographical information relevant to self-generated, internally focused thought, and which incorporates lower precuneus, superior and inferior anterior medial frontal regions, and posterior lateral parietal cortices) and the frontal-parietal network (FPN, which is drawn on to buffer internal trains of thought against disruption by external stimuli, and which incorporates rostrolateral prefrontal cortex, middle frontal gyrus, anterior insula, dorsal anterior cingulate cortex, precuneus, and anterior inferior parietal lobule; Spreng et al., 2010). Anatomically situated between the DMN and the dorsal attention network (DAN), the FPN’s posited role is to collaborate selectively with two networks that are focused on internally- and externally-focused cognitions, respectively. This “switching” model predicts that internally focused cognitions will be associated with activation in elements of the DMN and FPN, whereas externally focused cognitions will be associated with activation in elements of the FPN and DAN.

Two studies to date have used fMRI to examine the brain activation associated with internal and external cognitions. Vanhaudenhuyse et al. (2011) reported a linear relation between activity in DMN-related regions and participants’ ratings of intensity of internal awareness. They randomly beeped participants in the scanner and had them rate their state of awareness by pressing one of four buttons labeled *strongly external*, *moderately external*, *moderately internal*, and *strongly internal*. The random beeps (and thence the ratings) were presented, on average, every 20 s (range 3–30 s). However, such a rapid, repetitive rating procedure may substantially interfere with the natural resting state. Furthermore, the procedure may place participants in a strongly internal stance—they are required to monitor their experience essentially continuously because they know that they will have to rate their internal state at some time in the next 3–30 s. As a result, it can be argued that participants in such a situation have one of two equally undesirable options: they can try to ignore their experiment-imposed internal stance when rating their internal/external state (perhaps this is what the authors intended them to do); or they can rate the internality of the (experiment-imposed) state (but Hurlburt et al., 2016, have suggested that states elicited on demand may be psychologically and neurophysiologically different from states that spontaneously occur). Furthermore, the Vanhaudenhuyse et al. (2011) study defined the constructs (with “external” defined as “the perception of environmental sensory stimuli” and “internal” as “all environmental stimuli-independent thoughts,” p. 570), but did not seek to overcome presuppositions that participants might have regarding such constructs. Hurlburt (Hurlburt, 2011; Caracciolo and Hurlburt, 2016) has claimed that such presuppositions are powerful.

A second study employing experience sampling during fMRI is that of Van Calster et al. (2017), who used a think-aloud procedure in conjunction with cued experience sampling to generate categories of experience for which neural activations were established in a separate sample. Results showed a fluctuation in DAN activity as a function of subjective reports of attentional control, suggesting that this network is involved in recruitment of attentional processes during spontaneous cognition. Four categories of experience were derived for use in the fMRI investigations: absence of experience (blankness of mind), perceptions, stimulus-dependent thoughts, and stimulus-independent thoughts. Drawbacks of this methodology include a long duration of sampled experience (10 s before the probe), a complex response methodology involving the use of multiple fingers, categorization on the basis of frequency (thus not allowing for idiographic descriptions), relatively frequent sampling (probes at intervals between 30 and 60 s), and, perhaps most importantly, the fact that the distinction between a “thought” and a “perception” was not defined in this study. Hurlburt (Hurlburt and Schwitzgebel, 2007) reported that people apply the word “thinking” or “thought” to an extraordinary variety of inner experience, ranging from inner speech to feelings to sensory awareness.

The methods used in such studies force a categorization of experience, implying that experience is a unitary phenomenon, so that any particular experience is capable of being assigned as focused either on the environment or on inner processes, but not both simultaneously. There is reason to question this unitary-experience assumption. William James (1901), in his pioneering examination of the nature of consciousness, noted that any particular moment of experience is a dynamic composite of multiple streams. James’s insight is borne out by a large body of phenomenological data showing that experience can take multiple foci simultaneously. Hurlburt (2011) has found that people who have multiple experience, as discovered by Descriptive Experience Sampling (DES), often (perhaps usually) have no inkling of its existence prior to the DES explorations.

The study that provides the substrate for the present analyses was motivated by these and other methodological concerns. In that resting-state fMRI study (Kühn et al., 2014a; Hurlburt et al., 2015, 2016), we investigated experience during the resting state in a situation that (a) minimizes the disturbance of the resting state; (b) draws on a methodology capable of doing justice to the multiplicity of experience; and (c) provides an effective strategy for bracketing presuppositions about the relevant constructs.

We employed descriptions produced by the DES method (Hurlburt, 1997, 2011; Hurlburt and Akhter, 2006; Hurlburt and Heavey, 2006), an iterative procedure whereby participants develop expertise in responding to random beeps by making notes on the experience that was ongoing immediately before the beep, and subsequently exploring these moments of experience in detail with an investigator. In previous work, we have shown that DES can fruitfully be integrated with fMRI (Kühn et al., 2014a), and that it can provide richer descriptions of experience in the resting state than have previously been possible (Hurlburt et al., 2015). Hurlburt (2011) described the data elicited using this technique as providing a “high fidelity” account of inner experience, in the sense that DES aims to provide a clear and (largely) unbiased account of participants’ inner experiences.

Our participants were beeped four times during a 25 min scan (for an average inter-beep interval of about 400 s (20 times longer than Vanhaudenhuyse et al.’s (2011) 20 s average), thus substantially lessening the interference caused by the task. Furthermore, our aim was to acquire high-fidelity descriptions of experience, whatever that experience might be (in particular, neither our participants nor we were focused particularly on the internality/externality of their experience). Participants had been trained using an iterative procedure (Hurlburt, 2009, 2011) to aid in the bracketing of presuppositions and lessen misunderstandings between the participant and the investigators. In this way, we had generated 180 detailed descriptions of experiences that were collected from quasi-randomly selected (“beeped”) moments during resting-state scans, along with their associated fMRI data, all acquired within a standard resting-state paradigm as undisturbed by experimenter-imposed structure (that is, as “pristine”; Hurlburt, 2011) as the current state of the art allows.

The present article presents two re-analyses of these data with a specific focus on examining assumptions about the unitary nature of individual moments of experience. We selected an aspect of experience that has featured prominently in previous research, namely the internal/external model of mind-wandering as switching between internal and external foci. Both analyses began with detailed descriptions of samples of resting-state experience that had been generated by DES (Hurlburt et al., 2015). In the first analysis, we considered only those samples where experience was (according to our raters) indeed unitary and was unambiguously focused either internally or externally; then we asked how that internality or externality related to neural activations in those samples. This was essentially a replication of the earlier work by Vanhaudenhuyse et al. (2011) and Christoff et al. (2009), except that we did not *assume* that experience was unitary and either internal or external, but instead considered only those cases where the DES procedure (putatively) *established* that experience was indeed unitary and either internal or external. However, that analysis omitted samples that are potentially of more interest: those where there were two or more simultaneous streams of experience, one (or more) internal and another external; those where there was one stream of experience that had simultaneously an internal and an external focus; and those where internality/externality of experience was either ambiguous or irrelevant. We therefore conducted a second analysis that explored how the model accounted for those samples.

Our procedure was as follows. First, three independent raters coded our existing resting-state beeped-experience descriptions according to whether they were internally or externally focused (see Section “Materials and Methods” below). Limiting ourselves to experiences that could be unanimously classified, we contrasted (Analysis 1) the activations associated with experience samples classified as internally focused with those classified as externally focused, thus allowing a comparison of our results to the switching model of mind-wandering described above.

Then we recoded moments of experience for which there had not been unanimous agreement among the raters about an internal vs. external focus. Such situations were characterized by the division of experience between the internal and external worlds. In terms of the “switching” model of Spreng et al. (2010) and Smallwood et al. (2012; see above), the simplest way of accounting for such moments of experience would be in terms of simultaneous activation of *both* the DAN (focused on external experience) *and* the DMN (focused on internal experience). To assess this possibility, we attempted to predict (Analysis 2), from the fMRI activation data, internal–external ratings for the beeped experiences for which there had not been unanimous agreement. Such multiple or ambiguous experiences would be unclassifiable under standard categorical systems. Here, we draw on the richer nature of our experiential data (relative to previous studies) to test neural activation in such cases.

## Materials and Methods

As this study involves further analysis of data from a previous experiment, we highlight the method here and refer the reader to Hurlburt et al. (2016) for a complete description.

### Participants

Five native English-speaking participants who currently lived in Berlin participated on the basis of informed consent and with ethical committee approval according to the Declaration of Helsinki. All participants had normal or corrected-to-normal vision. No participant had a history of neurological, major medical, or psychiatric disorder, as determined by a questionnaire and the Mini-International Neuropsychiatric Interview (Sheehan et al., 1998; Ackenheil et al., 1999). The participants (three females, two males) had a mean age of 22.4 (ranging from 18 to 30) and all but one (male) were right-handed. The images from the left-handed subject were reversed, because we had evidence indicating that this participant activated right inferior frontal gyrus more strongly then left inferior frontal gyrus during a language generation task, i.e., the reverse of the pattern of lateralization typically seen in right-handers (Hurlburt et al., 2016).

### Measures

#### MRI Scanning

Images were collected on a 3T Magnetom Trio MRI scanner system (Siemens Medical Systems, Erlangen, Germany) using a 32-channel radio frequency head coil using a standard echo planar imaging (EPI) sequence. Images were obtained using a three-dimensional T1-weighted magnetization-prepared gradient-echo sequence (MPRAGE) based on the ADNI protocol^1^ (repetition time [TR] = 2,500 ms; echo time [TE] = 4.77 ms; TI = 1,100 ms, acquisition matrix = 256 × 256 × 176, flip angle = 7°; 1 mm × 1 mm × 1 mm voxel size). Functional images were collected using a T2^∗^-weighted EPI sequence sensitive to blood oxygen level dependent (BOLD) contrast (TR = 2,000 ms, TE = 30 ms, image matrix = 64 × 64, FOV = 216 mm, flip angle = 80°, voxel size 3 mm^3^ × 3 mm^3^ × 3 mm^3^, 36 axial slices).

fMRI data were analyzed using SPM8 software (Wellcome Department of Cognitive Neurology, London, United Kingdom), with commonly used preprocessing steps including slice time and motion correction, coregistration, normalization, and smoothing (see Kühn et al., 2014a, for details).

#### Descriptive Experience Sampling

Descriptive experience sampling was performed as described in Hurlburt and Heavey (2006), Hurlburt (2011), and elsewhere. DES is primarily an idiographic procedure, aiming at high fidelity apprehensions of inner experience phenomena regardless of whether those phenomena are common across individuals or idiosyncratic to particular individuals. The method produces a written description of each experience of each individual at each random beep.

### Procedure

Each participant was iteratively trained in 4 days of DES sampling in the participant’s natural environment (see Hurlburt et al., 2016, for a full description). We then continued DES sampling in nine 25-min sessions in the scanner with resting-state instructions: “Please relax, without falling asleep and do keep your eyes open.” In each session at four quasi-random times, the participant received a DES beep through a headphone. Immediately following each session, the participant participated in a DES expositional interview conducted by RH and at least one and as many as four additional interviewers, usually including SK and sometimes CF or BA-D.

Within 24 h of each DES expositional interview, one of the interviewers wrote a description of each of that day’s samples. These descriptions were then circulated to the others for comment, with any disagreement resolved or left as an explicit disagreement (see Hurlburt et al., 2017, for a discussion of this procedure), usually within 48 h of the original interview. It is these descriptions that serve as the starting point for the present study.

This sequence (25-min fMRI scan/four beeps followed by jotted notes/expositional interview/written description with commentary) was repeated eight more times, typically spread over 5 days, resulting in nine scanner sessions and 9 × 4 = 36 written descriptions of random samples of experience occurring in 9 × 25 = 225 min of fMRI scanning for each participant. In all, therefore, there were 5 × 36 = 180 sampled moments with associated written DES descriptions and time-locked fMRI data.

### Internal/External Coding of Experience Samples

Three of the authors (BA-D, CF, and RH) independently coded each of the 180 samples according to whether it could be confidently judged as involving either internally or externally generated experiences. The ratings were then compared across coders and three subsets were identified: internal (those samples for which all three raters unanimously agreed that the experience was internal; *n* = 65); external (those samples that were unanimously rated as external; *n* = 46); and non-consensus (those for which there had not been unanimous agreement; *n* = 69). Consideration of the non-consensus samples suggested that the main reason for the lack of consensus was that the samples contained both internal and external elements; this might provide an opportunity to examine neural activations when attention was focused neither exclusively internally nor exclusively externally.

The non-consensus samples were then subjected to forced-choice coding by the same raters. Each rater made a forced-choice judgment about whether each sample was predominantly internal (-1) vs. external (+1) and assigned a confidence score (between 1 and 10) for each judgment. These judgment and confidence scores were then multiplied together to form a continuous variable (from -10 if confidently internal to +10 if confidently external), and the mean taken across raters. This “composite” score, marking both internality/externality and confidence for each beeped experience, then became the dependent variable in the subsequent analyses. (See Table 1 for examples of samples unanimously classified as internal or external, and the Discussion for examples of non-consensus samples).

**Table 1:** Examples of sampled experiences classified as internal, external, and social.

Internal
Jack had been singing to himself the Beach Boys “Good Vibrations.” At the moment of the beep he is saying in his inner voice “good vibrations” in a declarative tone. Perhaps there is some musicality involved, but probably he is just saying what he had previously been singing.
Susan is visualizing very strongly a scene from yesterday: her boyfriend and his mother on a hillside next to the lake. She sees him in the shade, her in the sun, and (blurry) a sea of people around them. Before the beep she had been thinking that they look like monkeys, the way monkeys perch in family groups. Now she is somehow saying to herself in her own voice something like *they do look like monkeys*. There are words “floating around” but they don’t make full sentences. This is something like implied words rather than actually experienced words.

**External**

Lara is repeatedly blinking her eyes. She feels the blinking happen—that is, she does not experience herself as doing this blinking. Simultaneously she is hearing two tones coming from the scanner—one high, one low. She also sees her hand holding the pen, not noticing anything in particular. [Note that this is a multiple-strand experience, but all the strands are external.]
Otto is in a long blink, which he both feels in his eyes and sees blackness. He is simultaneously aware of his right arm, including the place that it touches the desk. He is simultaneously aware of his breathing—his chest rising, air in his nostrils, and the sound of the breathing.

**Social (note: all previously classified by at least one rater as internal)**

Lara is seeing herself saying “which a lot of them are very.” She sees herself incompletely detailed, but she sees herself in a third-person perspective from the front, upper torso wearing a white shirt and she has her hair up [today she’s wearing her hair down]. The words seem to be part of a larger sentence that preceded what she has written down and then would have gone on except for the interruption of the beeper. She is not sure who she is talking to, but the content is related to the beeper study.
Susan has been imagining herself bartering in Marrakesh, and she speaks to the seller in German: “*Was kostet das?*” Now she is innerly saying *silly* in a weird questioning tone that conveys her wonderment that she would speak in German to a person who probably doesn’t speak German.


### Whole-Brain Analysis

The statistical analyses were performed using a general linear model (GLM) approach. Regressors were built coding the (unanimous) internal and external events at the time of the audible beep. These vectors were convolved with a canonical hemodynamic response function (HRF) and its temporal derivatives.

Taking into account the standard 3–5 s delay in the hemodynamic response allowed us to model events in the brain in the seconds immediately prior to the moment of the beep. In a previous publication on the same fMRI data set (Hurlburt et al., 2016) we also tried modeling with stick functions 1 and 2 s before the beep onset and durations of 1–2 s for the “window” surveyed at each beep; these results were very similar, therefore we decided that modeling the event at the time of the beep is the most adequate procedure. Additionally, the six rigid body movement parameters were also included in the single subject GLM. Differential *t*-contrasts between (unanimous) internal vs. external events were calculated per subject and taken to group level analysis. On the second level, these differential *t*-contrast images were entered into a one-sample *t*-test. The whole-brain results were thresholded at *p* < 0.001, and to correct for multiple comparisons a significant effect was reported when the volume of the cluster was greater than the Monte Carlo simulation determined minimum cluster size above which the probability of type I error was <0.05 (Ward, 2000). The resulting maps were overlaid onto a normalized T1 weighted MNI template (colin27), and the coordinates reported correspond to the MNI coordinate system.

### ROI Analysis

From the regions resulting from the whole-brain contrasts we extracted percent signal changes for each region of interest (ROI) identified in the whole-brain analysis and for each individual and each sampled experience separately.

*K*-means clustering was then used to classify the non-consensus experiences based on percent signal changes in brain regions observed in the contrast of unanimous internal vs. external experiences. The objective of the *k*-means algorithm is to divide observations into clusters based on similarity of the cases (based on Euclidean distances) within each cluster so that the within-cluster sum of squares is minimized. In order to account for the fact that several data points were generated by the same subject and because there is, to our knowledge, no special repeated-measures approach to *k*-means clustering, we subtracted each subject’s mean from its respective ROI data. To explore whether the classes suggested by the *k*-means clustering algorithm related to our original internal/external coding, we ran a linear mixed-effects model predicting the composite rating from the *k*-means-derived clustering while accounting for repeated measures.

## Results

### Analysis 1: Contrasting Internal and External Samples

**FIGURE 1:**
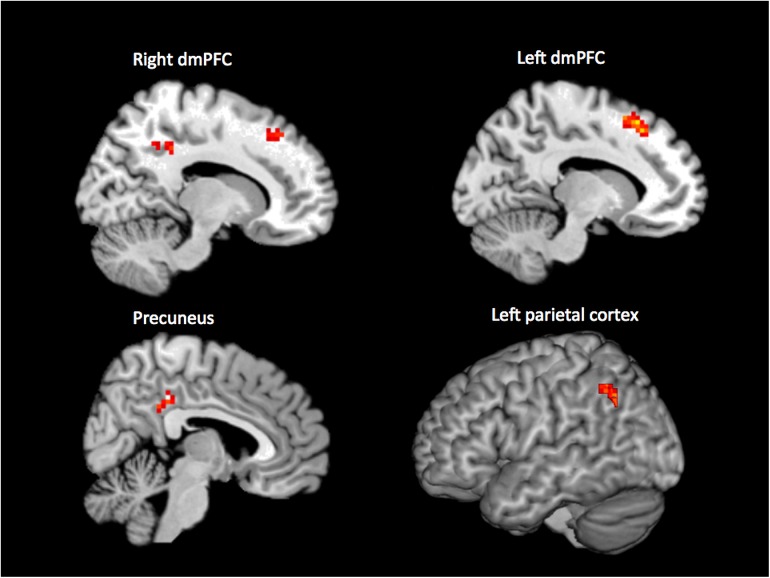
Results of the whole-brain contrast of unanimously classified internal vs. external samples (*p* < 0.001, cluster size corrected).

When performing an internal > external contrast on the (65 + 46 = ) 111 samples that were rated unanimously as being either internal or external, we observed activation in bilateral dorsomedial prefrontal cortex (dmPFC; 15, 32, 46; -12, 23, 52; BA 8, 9, 32), precuneus (6, -37, 37; BA 23), and left parietal cortex (-42, -61, 34; BA 7, 40) (*p* < 0.001, cluster corrected, Figure 1). For the reverse contrast (external > internal), no brain regions survived the threshold.

### Analysis 2: Predicting Forced-Choice Internal–External Classifications From the fMRI Data

We used a clustering approach to explore novel fMRI-data-driven ways to classify the (180–65–46 =) 69 non-consensus samples, treating the composite score (marking both internality/externality and confidence for each beeped experience) as a continuous variable in the regression. In order to predict these composite scores, we applied *k*-means clustering to the subject-demeaned percent signal changes extracted from each of the four ROIs identified in our original whole-brain analysis (right dmPFC, left dmPFC, precuneus, left parietal). We ran a linear mixed-effects model to predict the composite score, while accounting for the fact that multiple experiences came from the same subjects. The model was significant, *F*(1, 4.54) = 8.656, *p* = 0.036, indicating that the ROIs derived from those samples where internality/externality could be identified with confidence were also useful in using fMRI data to predict a continuous internality/externality rating in those other samples where our raters were less confident in their internality/externality classification.

### Subsidiary Analysis: *Post hoc* Coding of Social Content of “Internal” Experiences

Initial inspection of the regions highlighted by the internal > external contrast suggested that the region of activity in dmPFC was more dorsal than typical in a classic DMN pattern. One possible explanation is that activation in this region is an artifact of a confounding of social and internal/external aspects of experience. Our reasoning was as follows: internally directed experiences are likely to be sometimes social (involving other people) and sometimes non-social, because such experiences (thoughts, memories, daydreams, etc.) are not constrained by the physical environment of the scanner. In contrast, externally directed experiences in the scanner are highly likely to be non-social (about sounds and sensations in the scanner) and—given the physical constraints of the scanner, safety procedures around magnetic fields, etc.—unlikely to involve other people (unless, for example, people are visible in the scanner mirror). The dmPFC and precuneus are brain regions that have been implicated in social cognition (Kennedy and Adolphs, 2012; Mars et al., 2012; Schilbach et al., 2012). Our findings of internal > external dmPFC/precuneus activation might thus be an artifact of the social nature of internally directed experiences.

We therefore tested the possibility that the activation for internal experiences might reflect social processing. The same coders now rated the 65 unanimously classified internal experiences according to whether they were “social” (that is, involved other people). We coded events as social when at least one of the raters indicated that the event contained social elements. Of the 65 samples that had been unanimously classified as internal, 57 were coded as social (for examples, see Table 1); the remaining (65-57 =) 8 were designated “nonsocial.”

Next, we ran linear mixed-effect models with percent signal change in each of the ROIs as the dependent variable; as a predictor, we used a variable coding the social vs. non-social rating. In order to account for the fact that multiple experiences from each subject were entered into the analysis, we additionally added a subject variable. None of the linear mixed-effects models were significant, *F*(1, 66) < 1.58, *p* > 0.211, meaning that we did not find evidence that the activation in either bilateral dmPFC and/or precuneus can be explained by a systematic difference in the social content of experience.

## Discussion

The analyses presented here were designed to investigate how experience in the resting state can vary between internal, external, and multiple simultaneous streams. We used an existing neuroimaging dataset (Kühn et al., 2014a; Hurlburt et al., 2015, 2016) in which we have 180 quasi-random (“beeped”) DES apprehensions of participants’ first-person experience, each time-locked with their ongoing (MRI-measured) brain activity. In Analysis 1, we (i) considered those beeped experiences that were unanimously classified by three independent raters as being experientially internal (*n* = 65) or external (*n* = 46), (ii) modeled the corresponding BOLD response, and (iii) contrasted the modeled response for internal and external moments of experience. Whole-brain analysis revealed significantly higher brain activity in right dmPFC, left dmPFC, precuneus, and left parietal cortex for internal compared with external experiences.

All four brain regions identified in our internal > external contrast are part of the DMN (Buckner et al., 2008) and have been proposed as being deactivated during active task performance as compared to fixation (Gusnard and Raichle, 2001). Our finding thus supports existing models of resting-state cognition in demonstrating activation of the DMN during experiences that are internally generated. This support is important for two related reasons. First, the experiences we rated as being internally focused are indeed (as evidenced by the DES procedure and detailed in the phenomenological descriptions) experientially disconnected from the proximal environment; that is, they are genuinely (in terms of experience) “internal.” This constitutes a substantial advance over previous studies that have defined internality only in the negative, as self-reported failure to attend to an ongoing (external) cognitive task. In instances of such failure to attend, the participant’s attention might be drifting from one external focus (the task) to an equally external experience (e.g., to the noise of the scanner). In such cases, mind-wandering is not a *failure* of externality (resulting in a shift to internality) but rather a *shift* in externality from one external thing (the task) to another. To put it another way, mind-wandering has frequently been conceptualized as an involuntary allocation of external attention (e.g., McVay and Kane, 2010), rather than being intrinsically about internally focused experiences. In contrast, our internally focused experiences, as described by DES, are consistent with other characterizations of mind-wandering as allowing “freedom from immediacy” (Shadlen and Gold, 2004), liberating cognition from the pressure to respond to external stimuli in the environment.

Second, the shifts from external to internal and *vice versa* for our participants were events that took place in their natural environments (at least as natural as resting in a scanner can be). This constitutes a substantial advance over previous studies that have measured internal/external shifts in situations contrived by experimenters to be as explicitly boring as possible. In a report on the same sample that focused on a contrast between experimenter-elicited and spontaneous inner speech, Hurlburt et al. (2016) showed that the brain activations in on-demand situations may be different from activations in natural situations.

Thus our first analysis provides clear support for the idea that mind-wandering involves a decoupling of external stimuli, and that its phenomenology can be specifically linked to DMN activation. This is congruent with the findings of Vanhaudenhuyse et al. (2011) showing a linear relation between DMN-related activity and intensity of internal awareness.

However, only 111 of our 180 descriptions of experience (62%) could be unanimously classified into one of the two categories of internally oriented or externally oriented experience. The remaining 69 descriptions (38%) included multiple trains of thought (some internal, some external), elements of both internally and externally guided attention within unitary experiences, or experiences that were ambiguous or otherwise impossible to specify with regard to internality or externality. Such experiences are generally not contemplated by theories of mind-wandering.

Rather than eliminating these samples from the analysis or simply treating them as contributing to error variance (as must other analyses), we were able to draw on the phenomenological richness of DES reports to examine neural activations when attention was focused neither exclusively internally nor exclusively externally. In Analysis 2, we accordingly reversed the direction of explanation between levels of analysis. Rather than beginning with phenomenological descriptions and using them to characterize associated brain processes, we began with the regions of interest resulting from the whole-brain contrasts for our unanimously coded samples and used them to examine whether we could predict the phenomenology of the remaining samples for which there was not unanimous agreement. The model was successful in predicting our raters’ composite ratings (accounting both for forced-choice categorizations and for confidence) on the basis of the neural data. We have thus shown that the complex, multiple nature of spontaneous human experience described by William James and others can be brought into scientific inquiry. Specifically, we were able to link neural activations identified in established models of resting-state cognition to the phenomenological richness of complex, multi-layered moments of experience that would not have been amenable to standard mind-wandering methodologies.

Before considering the wider implications of our findings, we return to the observation that our dmPFC cluster was more dorsal than the dmPFC areas typically associated with the DMN. Because the localization is within the proximity of brain regions reported in social cognition (Schilbach et al., 2012), we conducted a control analysis (Section “Subsidiary Analysis: *Post-hoc* Coding of Social Content of “Internal” Experiences”), motivated by the observed activations and theoretical considerations, to exclude the possibility that the activation difference was an artifact of the likely greater sociality of internal vs. external experiences when sampled in an MRI environment. Our analysis suggested that this was not a likely explanation: the social content of sampled experiences did not predict brain activity in dmPFC. An alternative explanation suggests that dmPFC may be involved in an active disengagement from stimuli. This speculation is based on the observation that dmPFC is involved in voluntary inhibition (Filevich et al., 2012). Although to date only a few studies have investigated these endogenously generated inhibitory processes, the idea has a long heritage (Libet et al., 1983; Descartes, 1989). Interestingly, several of these studies suggest an involvement of dmPFC in the process of voluntarily suppressing actions (Brass and Haggard, 2007; Kühn et al., 2009) and emotions (Kühn et al., 2014b) as well as cigarette craving (Brody et al., 2007), which has led to the notion that dmPFC may be involved in the exertion of self-control (Lynn et al., 2014). Extending that idea leads to the speculation that our stronger activation in dmPFC during internal experiences may reflect an active disengagement from the stimuli in the proximal external environment.

We also note that we report neural activation differences between the two classifications of experience based on an internal > external contrast. The opposite contrast, external > internal, showed no significant differences. We speculate that this pattern of findings may suggest that there is no unitary externally focused cognition—that various kinds of externally directed cognition will each involve different sensory modalities, each with its own cortical localizations (to the different sensory cortices), so that activations in different modality-specific regions will cancel out in an external > internal contrast. Replication of this analysis with greater statistical power to interpret the null finding would be desirable.

In comparison with other studies that have assessed mind-wandering using versions of an attention-to-task paradigm (e.g., Christoff et al., 2009), the present study is distinctive in tying neural activations in the resting state to specific phenomenology. We have argued elsewhere that the use of a method such as DES in examining the phenomenology of the resting state avoids many of the confounds that attend other mind-wandering paradigms (Hurlburt et al., 2015). Our aim was to effect a closer integration than previously possible between detailed phenomenology and characterizations of neural activation, of a kind that researchers in this field are increasingly recommending (Fell, 2013; Christoff et al., 2016). We were able to do this in two directions: from phenomenology to neural activation and *vice versa*, drawing on the ability of DES, through the phenomenological richness of its descriptions, to capture moments of experience focused exclusively on neither the internal nor the external environment.

It is also clear from the foregoing report that only about two-thirds (111/180) of experience samples could be unanimously coded as having either an internal or an external focus. This suggests that, phenomenologically, either the distinction between internal and external focus is not as clear-cut as the dominant “switching” model would suggest, or that our coding procedure was not entirely adequate. To sort this through, we provide here a few examples (using fictional names for our five participants) where a simple characterization either as “internal” or “external” would be problematic.

(1) How should the occurrence of multiple simultaneous experiences be dealt with? The switching model holds that, if an internal train is to exist, external trains must be suppressed. (See our point above about a possible explanation for our dmPFC activation.) Our use of DES, however, repeatedly shows that two or more disparate experiences can exist simultaneously in all combinations: entirely in the external domain, entirely in the internal domain, or distributed between external and internal. As an example of the latter, Jane was focused on the geometry of the scanner above her head, particularly on the distance between the mirror and the ceiling of the scanner (an external focus). Simultaneously she innerly saw the office where the DES interviews had taken place, as if she had been walking into the room. She saw the table and RH, the people behind him, the computer, and so on. This imaginary seeing is an internal focus.
[As an example of the coding method, one of the raters coded this description external (confidence = 3), whereas the other two coded it internal (confidence = 3 and 4). For Analysis 2, we used the mean of these three ratings = (+3–3–4)/3 = -1.33, i.e., internal with a low confidence rating.]
(2) Should the purposeful suppressing of an external experience be considered internal or external? For example, Otto was actively trying not to hear the noise of the scanner. That is, he was *not* merely automatically screening out the scanner noise (which he did successfully on other occasions). Instead, he was hearing the noise while at the same time actively trying not to attend to it. In one manner of speaking, Otto’s hearing the scanner is an external process—he hears the noise. But in another manner of speaking Otto is engaged in an internally created task—he is purposefully trying to inhibit the hearing.
(3) Should an internally guided action, undertaken because it produces an external (see #5) sensation, itself be considered internal or external? For example, Susan was purposefully running her thumb along a cut that had healed on her finger. She was focusing on the roughness of the cut (an external sensation), but was doing so to perceive the resulting sensation (an internal intention).
(4) Should attending to a specific absence in the external environment be considered internal or external? For example, Jack was attending to the triangular shape of the space between his thumbs (not to the thumbs themselves). The thumbs themselves could be said to be external (as objects in the external world), but the triangleness of the space between them could be said to be an internal creation, *not* an aspect of the external environment.
(5) Should interoceptive experiences, such as sensations in the body, be considered internal or external? For example, Lara felt an itch beneath her left ear. This could be said to be internal (it takes place within the skin) but it could also be said to be external: the sensation seems, on the basis of a somewhat Cartesian distinction between mind/brain and body, to be external to the experiencing being. In this study, we agreed to treat such experiences as external.

On the face of it, experiences such as these five are problematic for an external–internal switching model. One possible future research avenue is to consider whether the switching model can be modified to account for multiple streams of consciousness, incorporating (for example) both internally and externally generated cognitions. In such cases, one might expect to see DMN activation in the absence of suppression of external stimuli by the FPN. It is also possible that multiple streams of experience are subserved by a different neural architecture to the FPN, a possibility that could be tested by comparing neural signatures of samples containing single vs. multiple streams of experience (whether all internal, all external, or a combination). In addition, the point made above about the dmPFC might entail that multiple streams might involve reduced activation in the dmPFC region.

However, it must be recognized that such research avenues may be impossible to travel using the forced-choice button-press methods typically employed in scanner studies of experience. We think it unreasonable to suppose, for example, that Jane (of example #1 above) could be taught (within the parameters of a single laboratory session) to notice disparate simultaneous trains of experience (e.g., exteriorly examining while simultaneously innerly seeing), to examine each train for its internality/externality, and to push a button if the trains differed in their internality/externality. As a result, it seems to us that studies that seek to take seriously the potential for extending the (unitary) switching model will have to be willing to engage in some kind of at-the-beep reporting that is of sufficiently high fidelity that outside observers can rate its internality/externality. In part because of the need for training, such studies will be quite labor-intensive.

The present study has several limitations. Because of the labor-intensive nature of the study, the sample size was small, although a large amount of phenomenological and neuroscientific data was collected for each participant, meaning that we have an acceptable level of power for our contrasts of interest. Despite the relatively small number of participants, such in-depth data can be used to demonstrate both confirmatory and surprising results from the combination of DES and fMRI (e.g., Kühn et al., 2014a). Nonetheless, the possibility that there was some unknown peculiarity of our group of participants cannot be ruled out. Our dataset was not sufficiently large to allow us to separate out non-consensus samples that included multiple trains of thought (some internal, some external), elements of both internally and externally guided attention within single experiences, or experiences that were ambiguous or otherwise impossible to specify internality or externality.

Finally, our design did not enable us directly to investigate the dynamic process, presumably unfolding over time, of switching between internal and external streams, which is a key component of the switching model (particularly as it relates to the maintenance of a particular state of internal or external focus over time). If one were able to capture such moments of transition between modes of attention, other important hubs of resting-state networks might also be apparent in our data. For example, the anterior insula and anterior cingulate cortex have been proposed to control salience-driven attentional switches between the DMN and FPN during the resting state (Sridharan et al., 2008; Wang et al., 2018). We observed no activation in these regions, but if it were possible to code our experience samples for recent or potential shifts in attention (essentially, moderations in what is salient internally vs. externally) then recruitment of this additional “salience network” may have become apparent (Seeley et al., 2007).

## Conclusion

We conclude by asking what the application of the labor-intensive method of DES contributes to the cognitive neuroscience of resting-state cognition that simpler, quicker methodologies do not. We argued at the outset that understanding the complexities of resting-state cognition requires nuanced phenomenological descriptions of the kind that cannot be provided by probe methods or questionnaires (see also Hurlburt et al., 2017). In the present study, we show that methods such as DES have the potential to unpick some of that complexity. We have shown that a model involving simple switching between internal and external focus cannot account for the (relatively common, according to our data) states of experience where both kinds of cognition are simultaneously ongoing. More importantly, this study is the first to tie neural activations in the resting state to specific, detailed phenomenology. Furthermore, careful examination of that phenomenology (as in the five examples above) opens important perspectives on phenomena that need to be understood. Although demanding in terms of resources, this study’s use of DES thus identifies valuable questions for future research into the resting state and mind-wandering.

## Ethics Statement

This study was carried out in accordance with the recommendations of the German Psychological Society Ethics Committee with written informed consent from all subjects. All subjects gave written informed consent in accordance with the Declaration of Helsinki. The protocol was approved by the German Psychological Society Ethics Committee.

## Author Contributions

CF, BA-D, RH, and SK conceived and designed the experiments, performed the experiments, analyzed the data, contributed reagents, materials, and analysis tools, and wrote the paper.

## Conflict of Interest Statement

The authors declare that the research was conducted in the absence of any commercial or financial relationships that could be construed as a potential conflict of interest.

## Footnotes

1 www.adni-info.org
